# Examining correlates of treatment satisfaction for injectable insulin in type 2 diabetes: lessons learned from a clinical trial comparing biphasic and basal analogues

**DOI:** 10.1186/1477-7525-5-8

**Published:** 2007-02-07

**Authors:** Meryl Brod, David Cobden, Morten Lammert, Donald Bushnell, Philip Raskin

**Affiliations:** 1 The BROD GROUP, 219 Julia Ave., Mill Valley, CA 94941, USA; 2 Novo Nordisk Inc., 100 College Rd. West, Princeton, NJ, 08540, USA; 3 Novo Nordisk A/S, Global Development, Novo Allé, 2880 Bagsværd, Denmark; 4 Health Research Associates, Inc. 6505 216th St. SW, Suite 105, Mountlake Terrace, WA. 98043, USA; 5 University of Texas, Southwestern Medical School at Dallas, 5323 Harry Hines Blvd., Dallas, TX 25390, USA

## Abstract

**Background:**

Successfully managing diabetes is a complex process that includes addressing issues of drug efficacy, safety and treatment satisfaction. Additionally, the combined impact of patient/disease characteristics and treatment outcomes on treatment satisfaction is not well understood. The purpose of this study was to examine the impact of age, weight, gender, co-morbid conditions, diabetes history, treatment burden, efficacy (HbA_1c_) and side effects (weight gain, hypoglycemic events) on patients' appraisal of treatment satisfaction using linear regression models.

**Methods:**

Data from a multi-center, randomized clinical trial comparing the efficacy/safety of biphasic insulin aspart 70/30 (BIAsp 70/30) vs. glargine (Glar) among insulin naïve type 2 patients were analyzed. Subjects were between ages 18–75, with baseline HbA_1c _> 8% and BMI ≤ 40 kg/m^2 ^(N = 233). Treatment satisfaction was assessed by the Insulin Treatment Satisfaction Questionnaire (ITSQ).

**Results:**

When factors were examined independently, multiple significant relationships (age, co-morbidity, hypoglycemic events, and weight gain) with overall and/or domains of treatment satisfaction were found. However, when all significant relationships were examined together, only neuropathy, treatment efficacy, and number of hypoglycemic events maintained their previous significance.

**Conclusion:**

By examining predictors independently, significant relationships were identified. However, not all findings remained significant when examined in combination with each other. Thus, to more accurately characterize the impact of factors on treatment satisfaction, a more comprehensive approach may be necessary. By improving patient treatment satisfaction, the efficacy of treatments, as well as critical treatment outcomes such as compliance and cost of care should be improved.

## Background

Successfully managing diabetes is a complex process and must address not only issues of drug efficacy and safety but also treatment satisfaction. Weaver and colleagues [1] defined treatment satisfaction as the patient's view of the treatment process and its associated outcomes based on predefined criteria. Treatment satisfaction is a key patient-reported outcome as it has been found to impact patient compliance [2,3], cost of care [4,5], and self-management behaviors [6]. Treatment satisfaction has also been shown to significantly differ between the array of drug treatment options available to clinicians for a given patient [7,8]. It may at times be more sensitive to change than quality of life, and has the ability to distinguish treatments with equivalent efficacy [9]. Persons with diabetes who perceive injecting insulin as burdensome may experience more negative health outcomes [10], whereas patients who are satisfied with their treatments are more likely to maintain positive physical and psychological health [11]. Understanding the elements of treatment satisfaction for diabetes is especially important, given the ever increasing range of treatment options for diabetes patients as treatment satisfaction expectations have been shown to be dynamic and changing with the introduction of new therapies [12].

Overall treatment satisfaction in diabetes consists of the patient's appraisal of 3 main treatment-related parameters: side effects, burden/inconvenience, and efficacy [13,14]. In diabetes, appraisal of side effects may predominantly include factors such as weight gain or the occurrence of hypoglycemic events. Appraisal of inconvenience or burden may take into consideration number of injections or type of delivery device. The gold standard for assessing efficacy is generally considered to be improvement in HbA_1c _level, although it is unclear if diabetes patients are aware of their exact values when not enrolled in a clinical trial. Additionally, patient and disease characteristics such as age, gender, time with diabetes, and prevalence and incidence of co-morbid conditions may act as mediators that influence these appraisals.

Static factors such as patient and disease characteristics are generally constant or fixed at any given point in time. Characteristics such as age, gender, level of education, ethnicity and duration of diabetes have been shown to account for some of the differences in patients' treatment satisfaction with their diabetes care when provided by endocrinologists compared to generalists [15]. However, data on the relationship of these factors to treatment satisfaction is often times conflicting, and many significant relationships do not endure when examined in combination with other important factors. For example, studies have found that greater satisfaction is associated with decreasing age, while others have indicated that younger patients are less satisfied with their treatment [16-18]. The occurrence of diabetes-related complications has been found to be associated with lower treatment satisfaction with diabetes medication, however, this association was no longer found when age, therapy and HbA_1c _level were also taken into consideration [17]. Insulin-treated patients have been found to have significantly greater treatment satisfaction than non-insulin treated patients with diabetes, though this significant difference was also lost after adjustments were made for age, gender, body mass index and duration of diabetes [19].

When examining treatment outcome factors which influence the appraisal of treatment satisfaction in diabetes, the most universal finding is that treatment satisfaction has generally been shown to be greater for those treatments that are most efficacious [13,17,20]. Additionally, clinical wisdom assumes that patients prefer treatments with few injections and few side effects such as weight gain or hypoglycemic events, although the data supporting these assumptions is not extensive and is contradictory [1]. For treatments with equal efficacy and no significant differences in weight gain or occurrence of hypoglycemic events, significant differences in treatment satisfaction have not been found [21]. Despite significant improvement in treatment satisfaction after 7 months of efficacious treatment (improved HbA_1c_) with decreased hypoglycemic events and increased weight, a significant relationship between weight gain, HbA_1c _and treatment satisfaction has not been found [22]. Interestingly, fewer number of daily injections has been shown both to improve treatment satisfaction and to have no impact on treatment satisfaction [23,24]. Further, continuous subcutaneous insulin infusion has been shown to be both superior to multiple daily injections, as well as to have no impact in determining patient treatment satisfaction [25,26].

Thus, it remains unclear what part of our beliefs about the relationships between patient/disease characteristics, clinical wisdom and treatment satisfaction in diabetes is myth versus reality. The purpose of this study was to examine, in the context of a randomized clinical trial (RCT), the correlative relationship between treatment satisfaction, patient/disease characteristics, and key treatment outcomes that impact the appraisal of treatment satisfaction. By understanding the myths vs. the realities of these relationships, clinicians will be in a better position to identify patients at risk for decreased treatment satisfaction and tailor treatment plans to maximize treatment satisfaction. By improving patient treatment satisfaction, the efficacy of diabetes treatments as well as other critical treatment outcomes such as compliance and cost of care is likely to be improved.

## Methods

### Procedures

Baseline and end of study (28 weeks) data from the INITIATE trial, a multi-center (25 U.S. sites) open-label RCT comparing the efficacy and safety of twice-daily (BID) biphasic insulin aspart 70/30 (BIAsp 70/30) vs. once-daily, bedtime (QD) glargine (Glar) among insulin naïve type 2 patients failing oral medication were analyzed. Eligible subjects had type 2 diabetes inadequately treated, controlled on oral antidiabetic agents (OADS), were insulin naïve, and male or female between the ages of 18–75 with baseline HbA_1c _> 8% and BMI ≤ 40 kg/m^2^. Patients with a history of recurrent, severe hypoglycemia, hepatic or renal insufficiency, cardiac disease, acute or chronic metabolic acidosis, active proliferative retinopathy or intolerance to metformin were excluded from the study. The first phase of the study was a 4 week run-in period with metformin with patients with a fasting blood glucose level of ≤ 140 mg/dL, or any single value ≤ 170 mg/dL considered a run-in failure. Patients who successfully completed the run-in period were then randomized to receive equal doses of BIAsp 70/30 within 15 minutes before breakfast and dinner in addition to their current metformin or Glar at bedtime in addition to their current metformin treatment. Subjects were told to perform daily blood glucose self-monitoring before breakfast and dinner. Insulin doses were titrated weekly for the first 12 weeks, then every 2 weeks thereafter according to a pre-defined titration algorithm based on blood glucose levels for the 3 days preceding a visit. Post-randomization, subjects were seen in the office weekly for the first month, and then again at weeks 8, 12, 16, 20, 24 and 28. Phone visits were conducted for weeks in which there was no office visit. Patients in the INITIATE trial signed informed consent forms and the study complied with FDA Good Clinical Practices. The study protocol had IRB approval.

### Measures

The following data were derived from and/or generated during the trial and were used for the present analyses:

• Patient characteristics and diabetes history including age (as a continuous variable), sex, ethnic group, time with diabetes (at baseline as a continuous variable), body mass index (BMI) and co-morbid conditions (classified as diabetes-related condition and as total number of conditions). These data were collected at the baseline visit.

• The Insulin Treatment Satisfaction Questionnaire (ITSQ) [11]: a validated measure consisting of 22 items that comprehensively assess treatment satisfaction for persons with diabetes on insulin. The measure was developed as a patient-reported outcome based on interviews with patients and clinical experts regarding treatment satisfaction as well as information in the literature. Satisfactory factor structure and internal consistency as well as adequate test-retest reliability, construct and discriminant validity for the ITSQ have been demonstrated [11]. In addition to an Overall score, the items make up five domains of satisfaction: Inconvenience of Regimen (IR-5 items), Lifestyle Flexibility (LF-3 items), Glycemic Control (GC-3 items), Hypoglycemic Control (HC-5 items) and Insulin Delivery Device (DD-6 items). All items are scored on a seven-point Likert-like response scale ranging from "not at all" to "extremely" (as worded appropriately for item). Items in the Glycemic and Hypoglycemic Control subscales are worded so that the respondent is asked about the relevant symptoms of their control such that a clinical understanding of either is not required. The ITSQ is scored by transforming all items to a scale of 0–100 with the higher score indicating better treatment satisfaction. For each subscale, the sum score is divided by number of items. Missing values are imputed based on the mean of the non-missing items. The ITSQ was administered after 28 weeks of treatment.

• Diabetic treatment effect status assessed after 28 weeks of treatment:

- HbA_1c _level

- Hypoglycemic episodes classified as total number, by timing of event (classified as day or night), and by type of event (classified as symptomatic or not)

- BMI classified as BMI group (< 25 as normal, between 25 and 29.9 as overweight, and ≥ 30 as obese) and by absolute value change (classified as improved or worsened)

### Statistical strategy

According to an *a priori *statistical analysis plan, for each statistical test performed, the relationship between the factors of interest, overall satisfaction and satisfaction within each domain of the ITSQ was examined by linear regression with listwise entry (all variables entered as a block to test interaction with treatment satisfaction as the dependent variable). Statistical significance was considered to be achieved with a minimum P value of 0.05.

#### Examining the relationship between patient and disease characteristics and treatment satisfaction

First, in order to confirm successfully randomized cohorts, differences between treatment groups for baseline patient and disease characteristics were examined by ANOVA or chi square as appropriate for each characteristic.

Next, a listwise linear regression analysis was performed to examine baseline predictors of treatment satisfaction assessed at week 28. The model included all demographic and diabetes history variables collected at baseline (age, gender, ethnicity, duration of diabetes diagnosis, BMI, and number/type of diabetes-related co-morbid conditions). Independent linear regression models were assessed for each of the ITSQ subscales and the Overall score.

The relationship of total number of co-morbid conditions and treatment satisfaction (each subscale and Overall) was examined by Pearson correlations. Additionally, the relationship between types of diabetes-related co-morbid conditions and treatment satisfaction was examined by independent linear regression (listwise) analyses of the diabetes-related co-morbid conditions found in this sample and treatment satisfaction.

#### Examining the relationships between treatment outcomes and treatment satisfaction

The relationship between the number of minor hypoglycemic events and treatment satisfaction was assessed by Pearson correlation (2-tailed). Type of event (symptomatic or not) and time of event (day or night), cumulative over the treatment period, and treatment satisfaction were examined by linear regression analyses independently for each subscale and Overall ITSQ score.

The relationship between BMI change during treatment and treatment satisfaction (Overall and all subscales) was first examined by performing an ANOVA, controlling for baseline weight, using change in BMI group from baseline to week 28. An independent ANOVA, controlling for baseline weight, examined the relationship between absolute changes in BMI, comparing those who had improved BMI to those whose BMI had worsened over the 28 weeks.

The relationship between HbA_1c _levels at week 28 and treatment satisfaction was examined by Pearson correlation coefficients.

The relationship between treatment group and treatment satisfaction was examined by ANOVAs for the total ITSQ score and each of its domains.

#### Examining the combined impact of significant factors

Lastly, as outlined in the statistical analysis plan, to understand the larger picture of the impact of demographic and treatment effect variables on treatment satisfaction, a regression analysis was performed on all of the above factors that were found to have a significant relationship to treatment satisfaction at week 28. Variance and goodness-of-fit was examined by the R^2^.

## Results

### Sample description

A total of 233 patients with Type 2 diabetes, a slight majority of which were male (52.9%) and Caucasian (51.7%) with an average age of 52.15 (± 10.34) years, were randomized into the INITIATE study. Two hundred and nine (209) of these subjects had treatment effect data and 197 of these completed the ITSQ and were included in these analyses. The average duration of diabetes diagnosis was 8.29 years (± 5.24), and mean baseline HbA_1c _was 9.71% (± 1.47%).

### Statistical findings

#### INITIATE trial clinical results

The INITIATE trial demonstrated that patients receiving BIAsp 70/30 were significantly more likely to have achieved HbA_1c _targets vs. Glar (66% vs. 40% of patients to HbA_1c _< 7%, p < 0.01, and 42% vs. 28% of patients to HbA_1c _≤ 6.5%, p < 0.05, respectively), and significantly improving total HbA_1c _reduction (-0.43%; p < 0.01 between arms). Post-prandial glucose excursions at lunch and supper were also significantly more tightly controlled (p < 0.05), though an increase in minor hypoglycemia and weight gain occurred (both p < 0.05) [27].

#### Examining the relationship between patient and disease characteristics and treatment satisfaction

There were no significant differences found between treatment groups for any of the baseline patient or disease characteristics, indicating that randomization of subjects to treatment groups used in the present analyses was successful.

As shown in Table 1, presence of diabetes-related co-morbid conditions at baseline was significantly predictive of overall treatment satisfaction as well as for Lifestyle Flexibility and in the Hypoglycemic and Glycemic Control subscales. Age was also predictive of treatment satisfaction related to Lifestyle Flexibility.

**Table 1:** Impact of Demographics and Diabetes History on Insulin-related Treatment Satisfaction

	**ITSQ Overall (n = 197)**	**ITSQ IR (n = 196)**	**ITSQ LF (n = 196)**	**ITSQ HC (n = 197)**	**ITSQ GC (n = 197)**	**ITSQ DD (n = 197)**
	**Coefficient (t-ratio)**					
*Intercept*	72.42*** (6.54)	74.63*** (5.89)	68.90*** (4.95)	67.67*** (4.83)	82.53*** (6.32)	73.68*** (6.32)
Age	0.21 (1.51)	0.23 (1.43)	0.35* (2.00)	0.22 (1.24)	0.17 (1.05)	0.14 (0.92)
Gender (0 = M, 1 = F)	-2.30 (-0.87)	0.45 (0.15)	0.23 (0.07)	-6.42 (-1.92)	-4.43 (-1.43)	-1.95 (-0.70)
Ethnicity (1 = C, 0 = Other)	-0.99 (-0.36)	-0.19 (-0.06)	-2.80 (-0.81)	-1.44 (-0.42)	-2.35 (-0.73)	0.90 (0.31)
Length of time with diabetes (↑ longer)	-0.17 (-0.66)	-0.09 (-0.32)	-0.06 (-0.17)	-0.42 (-1.30)	-0.13 (-0.42)	-0.17 (-0.61)
Weight-BMI	0.17 (0.66)	0.01 (0.04)	-0.23 (-0.70)	0.50 (1.53)	0.10 (0.31)	0.23 (0.83)
Number of diabetes related co-morbid conditions	-3.93* (-2.15)	-2.33 (-1.10)	-5.68* (-2.46)	-5.08* (-2.19)	-5.48* (-2.54)	-1.93 (-1.00)
	F = 1.195	F = 0.538	F = 1.812	F = 1.918	F = 1.497	F = 0.534
	R^2 ^= 0.036	R^2 ^= 0.016	R^2 ^= 0.053	R^2 ^= 0.056	R^2 ^= 0.044	R^2 ^= 0.016
	Adj R^2 ^= 0.006	Adj R^2 ^= -0.014	Adj R^2 ^= 0.024	Adj R^2 ^= 0.027	Adj R^2 ^= 0.015	Adj R^2 ^= -0.014

• * p < 0.05, ** p < 0.01, *** p < 0.001

• IR = Inconvenience of Regime subscale, LF = Lifestyle Flexibility subscale, HC = Hypoglycemic Control subscale, GC = Glycemic Control subscale, DD = Device Delivery subscale

The number of total co-morbid conditions in the sample ranged from 0 to 7 with a mean of 1.70 (± 1.50) and a median of 1.00. The diabetes-related co-morbid conditions identified in this sample were retinopathy, nephropathy, neuropathy, and macro-angiopathy. There were no significant relationships between total number of co-morbid conditions and treatment satisfaction. However, neuropathy was a significant predictor of treatment satisfaction (overall and in all subscales), indicating that pain is broadly associated with satisfaction outcomes. One other noted predictor was retinopathy, which was significant for the Device Satisfaction subscale. The relationship between types of diabetes-related co-morbid condition and treatment satisfaction is shown in Table 2.

**Table 2:** Impact of Diabetic-related Co-morbid Conditions on Treatment Satisfaction

	**ITSQ Overall (n = 197)**	**ITSQ IR (n = 196)**	**ITSQ LF (n = 196)**	**ITSQ HC (n = 197)**	**ITSQ GC (n = 197)**	**ITSQ DD (n = 197)**
	**Coefficient (t-ratio)**					
*Intercept*	84.11*** (56.89)	87.24*** (51.36)	78.85*** (41.72)	81.75*** (42.67)	86.43*** (49.64)	84.79*** (54.58)
Retinopathy (N = 0, Y = 1)	7.29 (1.50)	8.94 (1.60)	0.95 (0.15)	2.31 (0.37)	10.17 (1.78)	10.90* (2.13)
Nephropathy (N = 0, Y = 1)	2.08 (0.40)	-0.61 (-0.10)	3.68 (0.55)	3.79 (0.56)	-2.57 (-0.42)	3.94 (0.71)
Neuropathy (N = 0, Y = 1)	-10.56*** (-3.53)	-8.81* (-2.54)	-12.42** (-3.22)	-10.80** (-2.79)	-13.43*** (-3.82)	-8.00* (-2.54)
Macro Angiopathy (N = 0, Y = 1)	-5.18 (-1.09)	-1.16 (-0.21)	-4.58 (-0.76)	-5.63 (-0.92)	-5.93 (-1.06)	-6.44 (-1.29)
	F = 3.622**	F = 1.888	F = 2.943*	F = 2.280	F = 4.281**	F = 2.789*
	R^2 ^= 0.069	R^2 ^= 0.037	R^2 ^= 0.057	R^2 ^= 0.044	R^2 ^= 0.080	R^2 ^= 0.054
	Adj R^2 ^= 0.050	Adj R^2 ^= -0.018	Adj R^2 ^= 0.038	Adj R^2 ^= 0.025	Adj R^2 ^= 0.062	Adj R^2 ^= -0.035

• * p < 0.05, ** p < 0.01, *** p < 0.001

• IR = Inconvenience of Regime subscale, LF = Lifestyle Flexibility subscale, HC = Hypoglycemic Control subscale, GC = Glycemic Control subscale, DD = Device Delivery subscale

#### Examining the relationships between treatment outcomes on treatment satisfaction

A total of 1,073 hypoglycemic events, of which 1,072 were minor events, were reported over the 28 weeks of treatment. Hence, the analysis is based on minor hypoglycemic events only. The number of minor hypoglycemic events per patient was found to be significantly associated only with the Hypoglycemic Control subscale (r = -0.263, p < 0.001). However, as shown in Table 3, the time of a hypoglycemic event was found to be highly predictive of overall treatment satisfaction and for each subscale, with daytime events resulting in decreased treatment satisfaction. There were no significant impacts for the type of hypoglycemic event.

**Table 3:** Impact of Hypoglycemic Events on Treatment Satisfaction

	**ITSQ Overall (n = 197)**	**ITSQ IR (n = 196)**	**ITSQ LF (n = 196)**	**ITSQ HC (n = 197)**	**ITSQ GC (n = 197)**	**ITSQ DD (n = 197)**
	**Coefficient (t-ratio)**					
*Intercept*	92.74*** (12.96)	96.72*** (12.54)	85.36*** (8.46)	86.42*** (8.50)	95.64*** (10.88)	96.55*** (12.63)
Time of event (1 = Active, 7 AM-11 PM; 0 = Sleeping, 11 PM-7 AM)	-5.47*** (-4.52)	-5.13*** (-4.52)	-10.63*** (-6.22)	-4.39** (-2.55)	-2.98** (-2.00)	-5.26*** (-4.06)
Type of event (1 = Symptomatic, 0 = Not symptomatic)	-11.18 (-1.56)	-7.73 (-1.00)	-6.54 (-0.65)	-15.02 (-1.47)	-12.39 (-1.41)	-12.25 (-1.60)
	F = 11.854***	F = 8.454***	F = 19.837***	F = 4.555***	F = 3.164***	F = 9.929***
	R^2 ^= 0.026	R^2 ^= 0.018	R^2 ^= 0.042	R^2 ^= 0.010	R^2 ^= 0.007	R^2 ^= 0.022
	Adj R^2 ^= 0.023	Adj R^2 ^= 0.016	Adj R^2 ^= 0.040	Adj R^2 ^= 0.008	Adj R^2 ^= 0.005	Adj R^2 ^= 0.019

• ** p < 0.01, *** p < 0.001

• IR = Inconvenience of Regime subscale, LF = Lifestyle Flexibility subscale, HC = Hypoglycemic Control subscale, GC = Glycemic Control subscale, DD = Device Delivery subscale

The majority of patients (81%) did not change BMI group (normal, overweight or obese) during the treatment period, and no significant relationships between BMI group change and treatment satisfaction were found. The absolute BMI change ranged from -2.57 (decrease) to +6.83 (mean 1.61 ± 1.62) with the majority of the population (85.5%) increasing in BMI levels. For those 14.5 % of patients who had a decrease in BMI, there was a systematic improvement in treatment satisfaction across all scores, although only the difference for the Lifestyle Flexibility subscale was significant (p < 0.05). The lack of statistical significance may be due to the small number of patients who did change BMI group power (19%).

Pearson correlation coefficients showed a significant relationship between HbA_1c _and treatment satisfaction. While associations were low, the relationship of HbA_1c _level to treatment satisfaction was significant for the Overall score (r = -0.16, p < 0.05), Inconvenience of Regimen (r = -0.15, p < 0.05), Glycemic Control (r = -0.23, p < 0.001), and Device Satisfaction (r = -0.17, p < 0. 05).

No significant differences were found in the total ITSQ score or any of the domains during comparisons between treatment groups, indicating in part the potential that an increase in number of daily injections (BID vs. QD) may not negatively affect treatment satisfaction.

#### Examining the combined impact of significant factors

As shown in Table 4, when all previously identified significant relationships were examined together, neuropathy continued to have the broadest impact on treatment satisfaction (Overall and in all 5 subscales). Improved treatment efficacy (HbA_1c_) maintained the previously identified significant impacts on overall treatment satisfaction and in 3 subscales. The number of hypoglycemic events remained significant only for the Hypoglycemic Control subscale, however the timing of the hypoglycemic event significantly impacted overall satisfaction and 3 subscales rather than the previous 5 (Glycemic Control and Inconvenience of Regimen were no longer significant). The impact of age, weight gain and the number of co-morbid conditions were no longer significant. The R^2 ^for the model was 0.123, suggesting that these combined factors account for 12% of possible variance of factors that may impact treatment satisfaction.

**Table 4:** Impact of Significant Treatment Variables on Treatment Satisfaction

	**ITSQ Overall (n = 197)**	**ITSQ IR (n = 196)**	**ITSQ LF (n = 196)**	**ITSQ HC (n = 197)**	**ITSQ GC (n = 197)**	**ITSQ DD (n = 197)**
	**Coefficient (t-ratio)**					
*Intercept*	108.93*** (13.06)	110.62*** (11.53)	62.46*** (7.52)	84.27*** (43.10)	124.26*** (12.73)	108.68*** (12.34)
Age			0.32* (2.04)			
Neuropathy (N = 0, Y = 1)	-11.91** (-2.79)	-7.97* (-2.38)	-11.65* (-2.14)	-12.36* (-2.26)	-13.42** (-2.69)	-8.63** (-2.79)
Retinopathy (N = 0, Y = 1)						9.36 (1.86)
Change in weight (BMI)			7.40 (1.69)			
Co-morbid conditions (number of diabetic related)	1.43 (0.55)		-0.22 (-0.01)	1.83 (0.56)	0.04 (0.01)	
HbA_1c _Level (week 28)	-3.29** (-2.88)	-3.19* (-2.43)			-5.17*** (-3.87)	-3.19** (-2.65)
Number of hypoglycemic events during day (active 7 am-11 pm)	-0.53** (-2.72)	-0.29 (-1.28)	-0.70** (-2.86)	-0.81** (-3.26)	-0.34 (-1.47)	-0.42* (-2.06)
Number of hypoglycemic events during night (sleep 11 pm-7 am)	0.29 (0.72)	0.53 (1.14)	0.71 (1.41)	-0.27 (-0.53)	0.15 (0.32)	0.36 (0.84)
	F = 65.351***	F = 3.177*	F = 4.571***	F = 5.657***	F = 6.093***	F = 4.027**
	R^2 ^= 0.123	R^2 ^= 0.062	R^2 ^= 0.125	R^2 ^= 0.103	R^2 ^= 0.138	R^2 ^= 0.095
	Adj R^2 ^= 0.100	Adj R^2 ^= 0.043	Adj R^2 ^= 0.098	Adj R^2 ^= 0.085	Adj R^2 ^= 0.115	Adj R^2 ^= 0.072

• * p < 0.05, ** p < 0.01, *** p < 0.001

• IR = Inconvenience of Regime subscale, LF = Lifestyle Flexibility subscale, HC = Hypoglycemic Control subscale, GC = Glycemic Control subscale, DD = Device Delivery subscale

## Discussion

Clinical wisdom and common sense would presume, and research has at times supported the idea, that there is a negative impact of unfavorable treatment characteristics, burden of treatment and side effects on patient-reported treatment satisfaction. However, we did not find a significant relationship between the number of minor hypoglycemic events, weight gain or treatments with 1 vs. 2 daily injections with overall treatment satisfaction. The relationship of weight gain to treatment satisfaction was restricted only to Lifestyle Flexibility. This significant finding may be due to the nature of the items in the Lifestyle Flexibility domain, as 2 of the 3 items in that subscale concern eating times and meals. The timing of the hypoglycemic event (minor hypoglycemic events that occurred during the day vs. nocturnal episodes) did have a significantly negative impact on overall treatment satisfaction. However, it should be noted that it remains unclear if daytime hypoglycemic events are also more "feared" or problematic for patients, as nighttime events may be more difficult to identify or respond to and if they are major events they may have a greater potential for serious health consequences. It may also be noteworthy that minor diurnal events may be more bothersome to daily functioning, and therefore more negatively influence treatment satisfaction. On the contrary, minor nocturnal events such as patients' activity levels are likely to be higher during daytime hours. As there was only 1 severe hypoglycemic event during the study, we were not able to examine the relationship between major events and treatment satisfaction.

Also, contrary to common wisdom, no significant differences in treatment satisfaction were found between treatment groups despite the fact that BIAsp 70/30 was administered twice a day (with a pen device) and Glar was administered once a day (with a syringe) as a fixed characteristic of the treatment arm. This finding suggests that number of daily injections may not impact overall treatment satisfaction. However, due to the design of this study, it is difficult to separate out the effect of the number of daily injections versus the delivery device itself. It may be that the increased number of daily injections required for BIAsp 70/30 is balanced by a reported preference for the pen device over syringe [28-30]. It may also be that having 1 vs. 2 injections per day is not as important a factor of treatment satisfaction as the distress experienced by patients going from being insulin naïve (0 injections per day) to having begun injections on a daily basis [31]. Given that there is evidence suggesting that BIAsp 70/30 is also efficacious with once-daily injections [32], additional research should continue to examine the impact of once-daily injections with a pen versus syringe on overall satisfaction, as well as specifically for device satisfaction.

Regarding the belief that treatment efficacy is the primary driver of treatment satisfaction, BIAsp 70/30 was found to have superior efficacy (improvement in HbA_1c_) to Glar in the INITIATE trial, evidence that is supported in a subsequent clinical trial [33]. Although there is no data available to confirm that patients knew their HbAlc levels, doses were regularly titrated based on blood glucose levels and patients self-monitored their blood glucose levels twice daily. Further, HbAlc level was found to be significantly related to Overall Satisfaction suggesting that subjects were aware of treatment efficacy either by information from the physician or by how they felt. Thus, it is highly likely that the patients were aware of their HbA_1c _values, and able to use this factor when appraising their treatment satisfaction. Despite superior efficacy, treatment satisfaction was equivalent between the 2 treatment groups. This finding may be explained by the Decisional Balance Model of Treatment Satisfaction, whereby Overall treatment satisfaction is determined by the balance between the positive value of treatment and the negative harms and inconveniences of the medication [3]. Thus, the appraisal of treatment satisfaction for a treatment such as BIAsp 70/30, with superior efficacy when compared to Glar, but increased minor hypoglycemia and weight gain, may be equivalent. The Balance Theory model and our findings in the present analysis are supported by those in recent trials where competing insulin preparations which demonstrate disparate side effect and efficacy profiles have caused non-significant differences in treatment satisfaction [34]. The Balance Theory model may also help explain the lack of significant differences in overall treatment satisfaction in pen vs. syringe device studies, even when there is clearly stated preference for the pen device, if treatments have equivalent efficacy and safety [28].

It should be noted that the ITSQ overall score is not a truly independent rating of overall treatment satisfaction as it is the sum of the subscale items rather than a separate item assessing overall satisfaction. Post-hoc analyses with this study data have shown that the correlations between subscales and the overall score are all above 0.77 and a regression analysis of subscales on the Overall score, found all subscales were significantly associated with the Overall score at the 0.01 level, indicating a balance of the subscale weights contributing to the Overall score. We believe that in order to fully understand the impact of variables on treatment satisfaction, the impact on both subscales and Overall treatment satisfaction should be identified. Understanding the relative weight of subscales in the overall assessment may be valuable as this can help identify subscales of greater importance given a particular treatment or patient characteristic. Future studies may find it helpful to include a truly independent measure of overall satisfaction to further understand the contribution of subscales to overall satisfaction.

In this study we have examined both "simple" regression models looking at similar types of factors (e.g. demographic and patient characteristics) as well as more "complex" multivariate models allowing us to test if significance of variables from the like factor model is maintained when "competing" against a wide spectrum of influences. This incremental approach to the importance of factors, provides a more compressive picture of these factors and allows use to refine our understanding of their influence treatment satisfaction. It should be noted that although statistically significant relationships were identified in the study, the amount of variance accounted for in some of the models was small to moderate. Further, as with all statistical significance, it is unclear what the relationship is between the statistical and clinical significance of the findings and it is often difficult to separate out the unique contributions of variables which may be related, such as age and co-morbidity. Older type 2 diabetes patients are more likely to have diabetes related co-morbid conditions and these conditions may negatively impact a patient's ability to self-manage their disease, creating a barrier to positive outcomes.

Thus, it is important to understand the unique contributions of each of these factors to overall as well as domains of satisfaction. For example, although age did not have a significant impact on Overall satisfaction, neuropathy, a painful diabetes related condition, was the strongest predictor for decreased treatment satisfaction overall and across subscales. Although it may be difficult for physicians to reduce neuropathy-related pain in diabetes, lessening of pain has been found to play only a partial role in explaining treatment satisfaction for people with chronic pain. Several studies have found that provider-patient interaction factors such as confidence, trust and positive communications are also important for improving satisfaction [35-37]. Thus, improving provider-patient relationships for persons with diabetes and neuropathy may help improve their treatment satisfaction in general and lead to improved outcomes. This study did not examine the impact of non-diabetes related co-morbid conditions on treatment satisfaction and we suggest that this relationship be examined in future studies.

Diabetes related etinopathy was also a significant indicator for the Device Delivery subscale, indicating that visual impairments and the sensory experience should be taken into consideration in choosing an insulin delivery device for a given patient. When pen devices have been compared head-to-head (FlexPen vs. Humalog Pen vs. syringe), significant improvements in patient preferences have been found in favor of the devices which improve the readability of the dose scale and user confidence [28,38]. The auditory feature of pen devices has also been shown to affect the patient's confidence in selecting the correct dose [39], suggesting that auditory impairments also be considered when exploring treatment options.

These findings may also help clinicians identify patients who are most at risk for poor treatment satisfaction, so that the clinicians can develop targeted treatment plans for these patients. Certainly, patients with neuropathy or frequent daytime hypoglycemic events should be targeted for a discussion regarding their potential dissatisfaction with treatment. Additionally, their compliance and diabetes self-management behavior, critical factors for diabetes treatment success, should be assessed to identify if these behaviors have been negatively affected by their dissatisfaction. Finally, although cost of treatment was not examined in this study, it should be noted that biphasic insulin has been projected to be cost-effective therapy vs. basal insulin alone for long-term treatment [40,41]. Given the superior efficacy and equivalent patient-reported treatment satisfaction between these treatments, it may be reasonable for clinicians to consider the estimated improvements in quality-adjusted life expectancy along with potential cost savings associated with complication reductions.

Understanding the complex and multidimensional nature of treatment satisfaction as well as the interaction between factors that may impact how persons with diabetes appraise their treatments is still evolving. This study has begun to examine factors that may impact these relationships; considerably more research will be required to further unravel the myths and the realties of treatment satisfaction in diabetes. There are some shortcomings of this study that should be noted so that future research can address these issues. First, although satisfaction with previous treatments may significantly impact satisfaction with new treatments [42], the reality is that it is often difficult or unfeasible to assess. The lack of baseline treatment satisfaction also prevents the assessment of responsiveness of a measure to differences in treatment. These methodological challenges present limitations both in the broader treatment satisfaction literature and in this study. As the subjects were insulin naïve at baseline, previous insulin treatment satisfaction could not be measured. However, the absence of any differences in baseline demographic or health characteristics in the sample in combination with the study entry criteria requiring all subjects to have failed OADs, suggests that baseline differences in treatment satisfaction would not have been significant. Furthermore, a 4 week run-in period prior to the addition of the study insulin ensured that all randomized subjects were stabilized on an identical OAD regimen at baseline. It is also known that RCT populations are generally not representative of the entire population. Therefore, it would be very informative to examine these relationships in a more representative diabetic population such as in a clinic-based study. Additionally, although this study examined potential baseline patient/disease characteristics and key treatment outcomes that may influence treatment satisfaction, data on other important ongoing treatment factors such as burden and compliance were not collected. There may also be other diabetes as well as non diabetes related co-morbid conditions not reported in this sample, which may impact treatment satisfaction. These additional influences should be considered when looking at the broader spectrum of variables that may or may not impact treatment satisfaction. Inclusion of these variables or other potential drivers of treatment satisfaction may have increased the amount of variance in predicting treatment satisfaction in this study.

## Conclusion

Using baseline and end of study treatment effect data from a randomized controlled trial comparing the efficacy of BIAsp 70/30 (administered BID via pen device) and Glar (administered QD via syringe), and by correlating it to measured responses in the ITSQ, we examined the relationship between treatment satisfaction, patient/disease characteristics, treatment outcomes, and treatment groups. By assessing these factors independently, certain significant relationships were identified. However, not all of these significant findings survived when examined in combination with each other. Thus, in order to more accurately characterize the impact of patient/disease characteristics and treatment outcomes on treatment satisfaction, a more comprehensive approach at capturing data on all potentially relevant variables is necessary. This approach can enhance our understanding of factors that exert enduring or broader impacts on treatment satisfaction.

The perfect drug for diabetes would improve efficacy with no side effects, and the perfect patient with diabetes would be otherwise healthy. However, given the real world, clinicians will need to balance the advantages and disadvantages of various treatments and patient types, and aim to more fully understand the myths and realities of patient-reported treatment satisfaction to identify the optimal treatment for a given patient.

## Competing interests

The study reported on within this article was sponsored by Novo Nordisk. Authors 2 and 3 are current employees of Novo Nordisk.

## Authors' contributions

MB, DB, MLT and PR participated in the study design and conceptualization of the project. MB, DB, DC participated in the data analysis planning and execution. All authors read and approved the manuscript

## References

[B1] Weaver M, Patrick DL, Markson LE, Martin D, Frederic I, Berger M (1997). Issues in the measurement of satisfaction with treatment. Am J Man Care.

[B2] Albrecht G, Hoogstraten J (1998). Satisfaction as determination of compliance. Community Dent Oral Epidemiol.

[B3] Atkinson MJ, Kumar R, Cappelleri JC, Hass SL (2005). Hierarchical construct validity of the treatment Satisfaction Questionnaire for Medication (TSQM Version ll) among outpatient pharmacy consumers. Value Health.

[B4] Menzin J, Langley-Hawthorne C, Friedman M, Boulanger L, Cavanaugh R (2001). Potential short-term economic benefits of improved glycemic control: a managed care perspective. Diabetes Care.

[B5] Hogan P, Dall T, Nikolov P, American Diabetes Association (2003). Economic Costs of Diabetes in the U.S. in 2002. Diabetes Care.

[B6] Anderson RT, Marrero D, Skovlund SE, Cramer J, Schwartz S (2003). Self-reported compliance with insulin injection therapy in subjects with type 1 and 2 diabetes. Diabetologia.

[B7] Rosenstock J, Cappelleri JC, Bolinder B, Gerber RA (2004). Patient satisfaction and glycemic control after 1 year with inhaled insulin (Exubera) in patients with type 1 or type 2 diabetes. Diabetes Care.

[B8] Clark CM, Snyder JW, Meek RL, Stutz LM, Parkin CG (2001). A systematic approach to risk stratification and intervention within a managed care environment improves diabetes outcomes and patient satisfaction. Diabetes Care.

[B9] Colman SS, Brod MI, Krishnamurthy A, Rowland CR, Jirgens KJ, Gomez-Mancilla B (2001). Treatment satisfaction, functional status and health related quality of life of patients with migraine randomly assigned to almotriptan or aumatriptan. Clin Ther.

[B10] Weijman I, Ros WJ, Rutten GE, Schaufeli WB, Schabracq MJ, Winnubst JA (2005). Frequency and perceived burden of diabetes self-management activities in employees with insulin-treated diabetes: relationships with health outcomes. Diabetes Res Clin Pract.

[B11] Anderson RT, Skovlund SE, Marrero D, Levine DW, Meadows K, Brod M, Balkrishnan R (2004). Development and validation of the insulin treatment satisfaction questionnaire. Clin Ther.

[B12] Testa M (2003). Improving diabetes therapy: improving satisfaction. Diabetes Voice.

[B13] Brod M, Skovlund SE, Wittrup-Jensen KU (2006). Measuring the impact of diabetes through patient report of treatment satisfaction, productivity and symptom experience. Qual Life Res.

[B14] Atkinson MJ, Sinha A, Hass SL, Colman SS, Kumar RN, Brod M, Rowland CR (2004). Validation of a general measure of treatment satisfaction, the Treatment Satisfaction Questionnaire for Medication (TSQM), using a national panel study of chronic disease. Health Qual Life Outcomes.

[B15] Greenfield S, Kaplan SH, Kahn R, Ninomiya J, Griffith JL (2002). Profiling care provided by different groups of physicians: effects of patient case-mix (bias) and physician-level clustering on quality assessment results. Ann Intern Med.

[B16] Cappelleri JC, Gerber RA, Kourides IA, Gelfand RA (2000). Development and factor analysis of a questionnaire to measure patient satisfaction with injected and inhaled insulin for type 1 diabetes. Diabetes Care.

[B17] Redekop WK, Koopmanschap MA, Stolk RP, Rutten GE, Wolffenbuttel BH, Niessen LW (2002). Health-related quality of life and treatment satisfaction in Dutch patients with Type 2 diabetes. Diabetes Care.

[B18] Taylor MD, Frier BM, Gold AE, Deary IJ, Edinburgh Prospective Diabetes Study (2003). Psychosocial factors and diabetes-related outcomes following diagnosis of type 1 diabetes in adults: the Edinburgh prospective diabetes study. Diabet Med.

[B19] Petterson T, Lee P, Hollis S, Young B, Newton P, Dornan T (1998). Well-being and treatment satisfaction in older people with diabetes. Diabetes Care.

[B20] Bech P, Moses R, Gomis R (2003). The effect of prandial glucose regulation with repaglinide on treatment satisfaction, wellbeing and health status in patients with pharmacotherapy naive Type 2 diabetes: a placebo-controlled, multicentre study. Qual Life Res.

[B21] Herman WH, Ilag LL, Johnson SJ, Martin CL, Sinding J, Al Harthi A, Plunkett CD, LaPorte FB, Burke R, Brown MB, Halter JB, Raskin P (2005). A clinical trial of continuous subcutaneous insulin infusion versus multiple daily injections in older adults with type 2 diabetes. Diabetes Care.

[B22] Wilson M, Moore MP, Lunt H (2004). Treatment satisfaction after commencement of insulin in Type 2 diabetes. Diabetes Res Clin Pract.

[B23] Brod M, Lammert M, Raskin P (2005). Comparison of treatment satisfaction of twice-daily BIAsp 70/30 with once-daily insulin glargine in patients with type 2 diabetes. Poster presented at the 65th Scientific Session of the American Diabetes Association Meeting – San Diego, California, 2005 of the American Diabetes Association Meeting – San Diego, California.

[B24] Bradley C, Plewe G, Kliebe-Frisch (2005). Treatment satisfaction with a basal insulin added to oral agents versus twice-daily premixed insulin alone in patients with Type 2 diabetes. Diabetes.

[B25] Raskin P, Bode PW, Marks JB, Hirsch IB, Weinstein RL, McGill JB, Peterson GE, Mudaliar SR, Reinhardt RR (2003). Continuous subcutaneious insulin infusion and multiple daily injection therapy are equally effective in type 2 diabetes: a randomized, parallel-group, 24 week study. Diabetes Care.

[B26] Hoogma RP, Spijker AJ, van Doorn-Scheele M, van Doorn TT, Michels RP, van Doorn RG, Levi M, Hoekstra JB (2004). Quality of Life and metabolic control in patients with diabetes mellitus type 1 treated by continuous subcutaneous insulin infusion of multiple daily insulin injections. Neth J Med.

[B27] Raskin P, Allen E, Hollander P, Lewin A, Gabbay RA, Hu P, Bode B, Garber A, INITIATE Study Group (2005). Initiating insulin therapy in type 2 diabetes. Diabetes Care.

[B28] Korytkowski M, Bell D, Jacobsen C, Suwannasari R, FlexPen Study Team (2003). A multicenter, randomized open-label comparative, two-period crossover trial of preference, efficacy, and safety profiles of a prefilled, disposable pen and conventional vial/syringe for insulin injection in patients with type 1 or 2 diabetes mellitus. Clin Ther.

[B29] Rubin RR, Peyrot M (2004). Quality of life, treatment satisfaction and treatment preference associated with use of a pen device delivering a pemixed 70/30 insulin aspart suspension (aspart protamine suspension/soluble aspart) versus alternative treatment strategies. Diabetes Care.

[B30] Summers KH, Szeinbach SL, Lenox SM (2004). Preference for insulin delivery systems among current users and nonusers. Clin Ther.

[B31] Peyrot M, Rubin RR, Lauritzen T, Skovlund SE, Snoek FJ, Matthews DR, Landgraf R, Kleinebreil L, The International DAWN Advisory Panel (2005). Resistance to insulin therapy among patients and providers: results of the cross-national Diabetes Attitudes, Wishes, and Needs (DAWN) study. Diabetes Care.

[B32] Garber AJ, Wahlen J, Wahl T, Bressler P, Braceras R, Allen E, Jain R (2006). Attainment of glycaemic goals in type 2 diabetes with once-twice, or thrice-daily dosing with biphasic insulin aspart 70/30 (The 1-2-3 study). Diabetes Obes Metab.

[B33] Kann P, Wascher T, Zackova V, Moeller J, Medding J, Szocs A, Mokan M, Mrevlje F, Regulshki M (2006). Starting insulin therapy in type 2 diabetes: twice-daily biphasic insulin Aspart 30 plus metformin versus once-daily insulin glargine plus glimepiride. Exp Clin Endocrinol Diabetes.

[B34] Goudswaard AN, Stolk RP, Zuithoff P, de Valk HW, Rutten GE (2004). Starting insulin in type 2 diabetes: continue oral hypoglycemic agents? A randomized trial in primary care. The J Fam Pract.

[B35] Shaw WS, Zaia A, Pransky G, Winters T, Patterson WB (2005). Perceptions of provider communication and patient satisfaction for treatment of acute low back pain. J Occup Environ Med.

[B36] Hirsh AT, Atchison JW, Berger JJ, Waxenberg LB, Lafayette-Lucey A, Bulcourf BB, Robinson ME (2005). Patient satisfaction with treatment for chronic pain: predictors and relationship to compliance. Clin J Pain.

[B37] McCracken LM, Klock PA, Mingay DJ, Asbury JK, Sinclair DM (1997). Assessment of satisfaction with treatment for chronic pain. J Pain Symptom Manage.

[B38] Niskanen L, Jensen EJ, Rastam J, Nygaard-Pedersen L, Erichsen K, Vora JP (2004). Randomized, multinational open-label, 2-period, crossover comparison of biphasic insulin aspart 30 and biphasic insulin lispor 25 and pen devices in adult patients with Type 2 diabetes mellitus. Clin Ther.

[B39] Asakura T, Seino H (2005). Assessment of dose selection attributes with audible notification in insulin pen devices. Diabetes Technol Ther.

[B40] Ray JA, Valentine J, Nicklasson L, Cobden D, Raskin P, Garber A, Palmer AJ (2007). Insulin therapy in type 2 diabetes patients failing oral agents: cost-effectiveness of biphaic insulin aspart 70/30 vs. insulin glargine in the US. Diabetes Obes Metab.

[B41] Valentine WJ, Palmer AJ, Lammert M, Nicklasson L, Foos V, Roze S (2005). Long-term clinical and cost outcomes of treatment with biphasic insulin aspart 30/70 versus insulin glargine in insulin naive type 2 diabetes patients: cost-effectiveness analysis in the UK setting. Curr Med Res Opin.

[B42] Hurwitz EL, Morgenstern, Yu F (2005). Satisfaction as a predictor of clinical outcomes among chiropractic and medical patients enrolled in the UCLA low back pain study. Spine.

